# A Systematic Review of Health Disparities in Chronic Rhinosinusitis in the United States

**DOI:** 10.1002/oto2.70163

**Published:** 2025-09-12

**Authors:** Russell A. Whitehead, Abdel R. Metwally, Evan A. Patel, Thomas Cyberski, Robin Powszok, Peter Filip, Peter Papagiannopoulos, Bobby A. Tajudeen, Pete S. Batra

**Affiliations:** ^1^ Department of Otolaryngology–Head and Neck Surgery Rush University Medical Center Chicago Illinois USA

**Keywords:** chronic sinusitis, gender, geographic region, health disparities, race, rhinology, sinus surgery, social determinants, socioeconomic status

## Abstract

**Objective:**

Literature describing disparities in chronic rhinosinusitis (CRS) often analyzes race, gender, or socioeconomic status (SES) in isolation. Analyses encompassing a comprehensive range of disparities remain lacking. We conducted a systematic review to provide a detailed characterization of the CRS disparity landscape.

**Data Sources:**

A systematic review was conducted in Covidence adhering to Preferred Reporting Items for Systematic Reviews and Meta‐analyses (PRISMA) guidelines. A search of PubMed/MEDLINE, CINAHL, Scopus, etc. was performed for literature published through September 2024.

**Review Methods:**

A total of 690 publications were identified and screened by two authors independently. In total, 26 ultimately met the inclusion criteria. Studies were classified by pertaining health disparity (race, gender, SES, age, and geographic region) and reported outcomes (incidence, severity, and treatment choice).

**Results:**

In total, 26 studies on CRS disparities were published from 2012 to 2024. 16 focused on SES, describing that lower SES was associated with reduced treatment adherence, resulting in poorer endoscopic findings and quality of life. 14 studies examined racial/ethnic disparities. Hispanic patients were more symptomatic than non‐Hispanic patients, whereas black patients had fewer health visits, leading to worse outcomes. Other studies discussed the impact of gender, age, and/or geographic region (n = 9, 4, 4, respectively). Findings included higher symptom burden among female patients and higher CRS incidence in regions of air pollution. Only three studies proposed solutions to disparities.

**Conclusion:**

Most literature on CRS disparities describes the influence of SES and race on disease presentation and progression. Other disparities related to gender, age, and geographic region were identified. Further research should uncover root causes and propose detailed solutions to advance equitable care in CRS.

Chronic rhinosinusitis (CRS) impacts between 1% and 5% of the United States population and has been shown to be associated with a multitude of health disparities that affect resource utilization, disease severity, and treatment outcomes.^1^, ^2^, ^3^, ^4^, ^5^, ^6^ The Social Determinants of Health (SDOH) are nonmedical factors proven to affect patient outcomes, including healthcare access, economic stability, education, community context, and the built environment.^7^ A growing body of literature describes the impact of SDOH on otolaryngology care; specifically, outcomes in head and neck cancer, hearing loss, reflux, and CRS.^8^, ^9^, ^10^, ^11^, ^12^, ^13^, ^14^ Recognizing the impact of SDOH enables providers to deliver more personalized, effective treatments and improve overall health equity.

Current literature describing disparities in CRS often analyzes the impact of race, gender, or socioeconomic status (SES) in isolation.^15^, ^16^, ^17^ For example, several studies have described how non‐Hispanic black (NHB) patients with CRS have poorer outcomes after endoscopic sinus surgery (ESS) compared to non‐Hispanic white (NHW) and Hispanic patients.^6^, ^16^, ^18^ NHB patients have also been shown to be more likely to present with more severe disease at initial consultation.^19^ Additionally, lower SES has been shown to predict increased exposure to air pollution and increased severity of CRS, quantified by increased steroid treatments.^17^ Despite these studies, analyses encompassing a comprehensive range of disparities in CRS remain lacking. Systematic reviews of health disparities in otolaryngology remain limited with head and neck cancers being the one area demonstrating a more robust body of literature.^8^, ^10^ This paucity of literature underscores the need for a comprehensive understanding of health disparities in CRS to improve equity across patient populations.

Therefore, the objective of this systematic review is to thematically characterize existing research on the impact of sociodemographic factors on CRS patients in the United States. This aims to provide transparency to the current state of literature and elucidate its findings and implications.

## Methods

**Figure 1:**
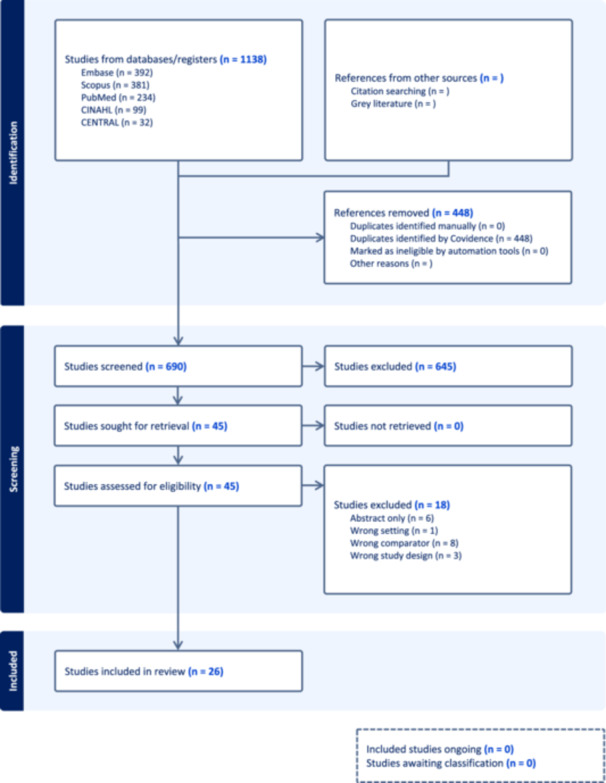
Preferred Reporting Items for Systematic Reviews and Meta‐analyses flow diagram of articles included in systematic review.

A systematic review was conducted in Covidence (a web‐based collaboration software platform that streamlines the production of systematic literature reviews^20^) following Preferred Reporting Items for Systematic Reviews and Meta‐analyses (PRISMA) guidelines and was not registered in PROSPERO. A comprehensive literature search was developed by the authors and run by two experienced medical librarians on September 3, 2024. A search of PubMed/MEDLINE, Embase, CINAHL, Scopus, and the Cochrane CENTRAL Register of Controlled Trials was performed for literature published from database inception through September 2024 evaluating health disparities in CRS. Both controlled vocabularies (eg, MeSH terms) and keywords in the title or abstract fields were searched. Additionally, a hand search was conducted of the reference lists of selected articles. This yielded the initial identification of 1138 publications. Publications were then screened by two individual reviewers (R.A.W. and A.R.M.). Inclusion criteria were assessed by each author, and inconsistencies were resolved by consensus. Full texts were examined for relevance. Figure 1 displays exported PRISMA data. Supplemental File S1, available online, contains the full search string including MeSH terms and keywords used for database search.

Studies that were included in this analysis were focused on health disparities in CRS in the United States, including the impact of SES, race/ethnicity, gender, age, or geographic region. SES was generally equated to median household income or insurance status (private insurance, Medicare, and Medicaid). Racial groupings were primarily NHW, NHB, Hispanic, and to a lesser extent, Asian patients due to their relative frequency in the literature. When discussing differences between male and female patients, a majority of studies utilized the term “gender” as opposed to “sex”; therefore, the rhetoric of this review reflects that.

Studies that were excluded were written in a non‐English language, in an international setting, submitted as conference abstracts, were non‐accessible, studies without a health disparity aim/focus, case reports, and case series.

Given the marked heterogeneity of included study designs within, including retrospective cohorts, cross‐sectional analyses, and database studies, it proved methodologically challenging to apply a single validated risk‐of‐bias tool in a meaningful way. Many available tools are narrowly designed for specific study types and do not capture the broader context and complexity often inherent in health disparities research. The application of multiple tools across different subsets of studies would require significant interpretive judgments that likely would not improve the utility of the review.

The following datapoints were collected on each study and depicted in Table 1: title, authors, publication year, study design, study population (ie, SES, race/ethnicity, gender, age, and geographic region), study outcomes (ie, disease severity, incidence/prevalence, poor health behaviors, treatment modality, and pathophysiology of disease), and future steps/interventions offered. Disease severity was quantified by endoscopic exam scores and subjective symptom scores. Poor health behaviors were primarily described as inadequate adherence to medication regimens and follow‐up appointments. Treatment modality was generally defined as medical or surgical treatment. Pathophysiology of disease was referred to as differences in sinus anatomy between patients.

**Table 1:** Study Characteristics

Author	Year	Study design	Health disparity population					Outcomes discussed					Future steps/interventions offered
			Socioeconomic status	Race/ethnicity	Gender	Age	Geographic region	Disease severity	Incidence/prevalence	Poor health behaviors	Treatment modality/outcomes	Pathophysiology of disease	
Soler	2012	Retrospective	−	+	−	−	−	+	−	+	−	−	−
Smith	2013	Retrospective	+	+	+	−	+	−	+	−	−	−	+
Samuelson	2017	Retrospective	+	+	+	+	−	−	+	−	+	−	−
Bergmark	2018	Prospective	+	+	−	−	−	+	−	−	−	−	−
Beswick	2019	Prospective	+	−	+	+	−	+	−	−	−	−	−
Beswick	2019	Prospective	+	−	+	−	−	−	+	−	−	−	−
Phillips	2019	Prospective	−	−	+	−	−	+	−	−	−	−	−
Duerson	2019	Retrospective	+	+	−	+	−	+	+	+	−	−	−
Shen	2020	Retrospective	+	+	−	−	−	+	−	−	−	−	−
Levine	2021	Retrospective	−	+	−	−	−	+	−	−	−	−	−
Pandrangi	2021	Cross‐sectional	+	−	−	−	−	−	+	+	+	−	+
Reyes Orozco	2022	Retrospective	−	+	−	−	−	−	−	+	+	−	−
Velasquez	2022	Retrospective	+	−	−	−	+	+	+	−	−	−	−
Kim	2022	Retrospective	−	+	−	−	−	+	+	−	−	−	−
Konsur	2022	Prospective	−	+	−	−	−	+	+	+	+	−	−
Konsur	2022	Prospective	+	−	−	−	−	+	+	+	+	−	−
Tam	2023	Retrospective	−	+	−	−	−	+	−	−	+	−	−
Asokan	2023	Prospective	−	−	+	−	−	+	−	−	+	−	−
Peterson	2023	Systematic review	+	+	−	−	−	+	−	−	−	−	−
Behnke	2024	Systematic review	−	−	+	−	−	+	−	−	+	+	+
Gill	2024	Prospective	+	−	−	−	+	+	−	−	−	−	−
Ziltzer	2024	Cross‐sectional	+	−	−	−	−	+	+	−	−	−	−
Han	2024	Prospective	+	+	+	+	−	+	+	−	−	−	−
Hentati	2024	Retrospective	+	+	−	−	−	+	+	−	+	−	−
Wang	2024	Prospective	+	−	−	−	+	+	−	−	+	−	−
Ryan	2024	Systematic review	−	−	+	−	−	+	−	−	+	−	−

## Results

### Summary of Health Disparities

**Figure 2:**
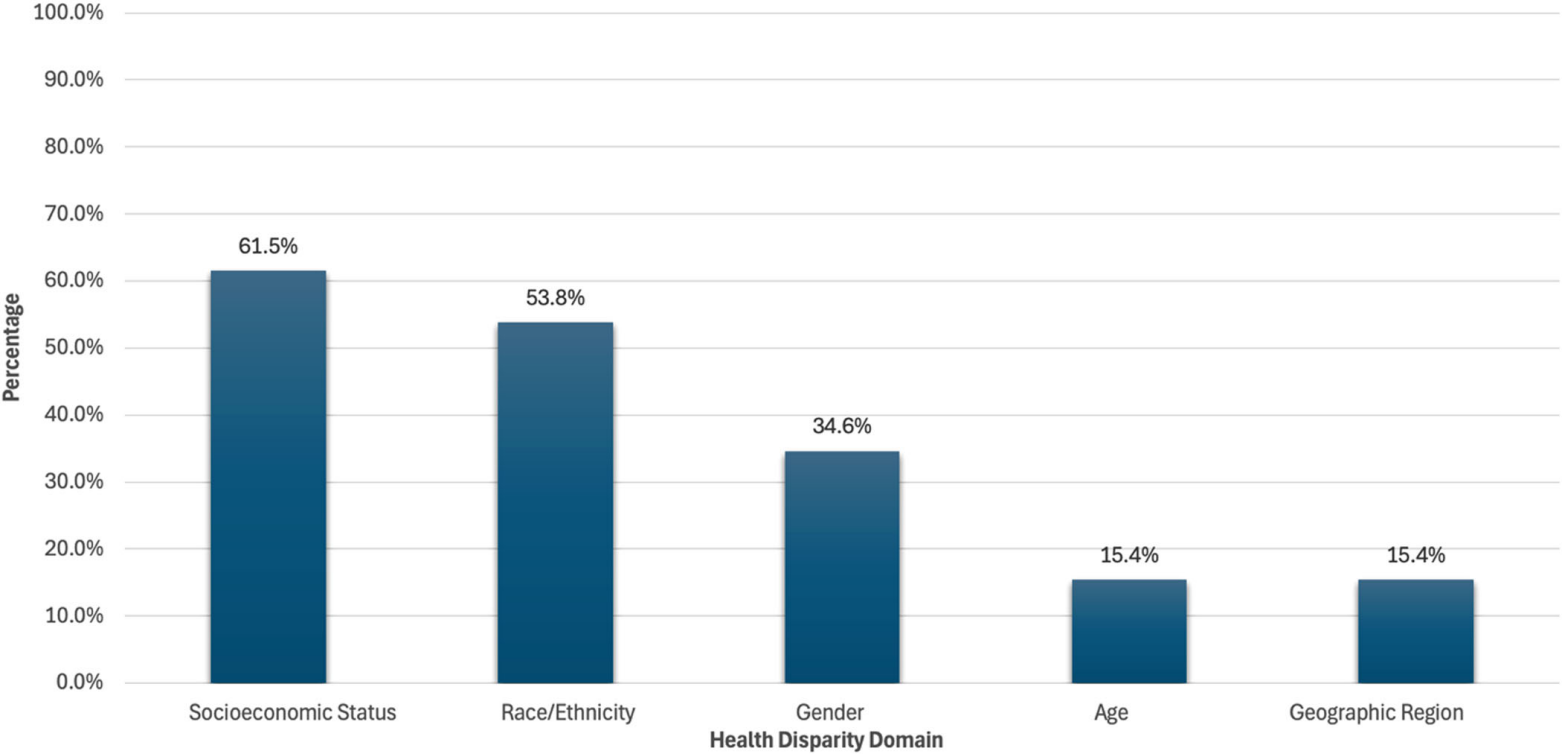
Breakdown of health disparities discussed in existing literature.

In total, 26 studies on CRS disparities were published between the years of 2012 and 2024. The majority were retrospective studies (11), followed by prospective (10), systematic reviews (3), and cross‐sectional studies (2). Many of the studies focused on SES as the source of disparity (16), followed by race/ethnicity (14), gender (9), age (4), and geographic region (4) (Figure 2).

**Figure 3:**
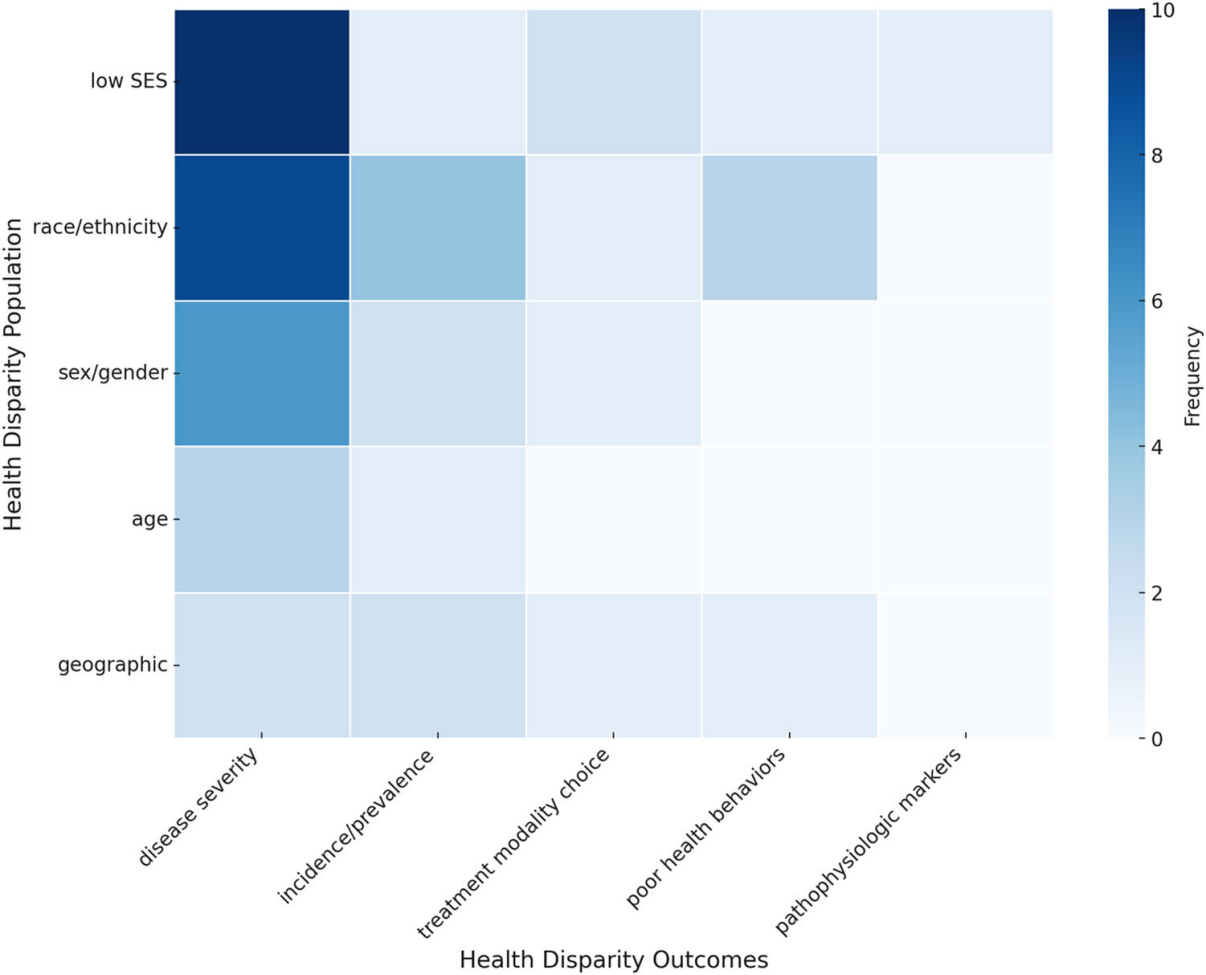
Heatmap of health disparity populations versus outcomes. Articles from current literature that discuss health disparity populations and outcomes including disease severity, incidence/prevalence, treatment modality choice, poor health behaviors, and disease pathophysiology. SES, socioeconomic status.

The outcomes discussed in most studies included the impact of health disparities on disease severity (21), incidence/prevalence (12), treatment modality/outcomes (11), poor health behaviors (6), and pathophysiology of disease (1) (Figure 3).

Only 3 of 26 studies (11.5%) suggested future directions or ideas for further research to improve disparities.

### Socioeconomic Status

Sixteen studies (61.5%) focused on SES, including the impact of income and insurance status. One study found that adult CRS patients seeking tertiary rhinology services were more likely to have a higher median incomes or private insurance compared to the general hospital population (average patient seen at that hospital).^21^ Additionally, separate studies found that patients with higher income or private insurance significantly improved their work productivity (required less time off) and perceived health status post‐ESS.^6^, ^22^ In multiple studies, lower income and unhoused status were associated with more severe symptoms at initial consultation, reduced likelihood of follow‐ups, and higher likelihood of being treated at public hospitals.^4^, ^15^, ^18^, ^23^, ^24^ Financial insecurity was associated with treatment noncompliance and requests for lower‐cost medications; decisions patients potentially made to decrease medical expenses.^25^ Patients who were more socioeconomically disadvantaged and those with Medicaid insurance were reported to face higher exposure to air pollution, more severe CRS symptoms, and required increased systemic steroid treatment.^15^, ^17^, ^26^ In a cohort of patients with cystic fibrosis and CRS, Medicaid/Medicare insurance correlated with more severe pathology on endoscopy than those with private insurance.^27^ In contrast, two studies showed that SES and insurance status had no significant effect on initial CRS severity, Sino‐Nasal Outcome Test‐22 (SNOT‐22) scores, quality‐of‐life (QoL) scores, or treatment receipt.^4^, ^28^

In the pediatric CRS population, patients were significantly more likely to have private insurance compared to the general hospital population.^29^ Privately insured pediatric patients were more likely to have received prior allergy referrals. Finally, lower SES was associated with decreased QoL.^11^, ^29^ However, SES and insurance status were not predictive of whether pediatric patients received medical versus surgical therapy.^29^

### Race/Ethnicity

Fourteen studies (53.8%) discussed how patient race/ethnicity contributed to health disparities in CRS. It was reported that adult and pediatric patients with CRS who presented to a tertiary rhinology center were more likely to be NHW compared to the general hospital population.^21^, ^29^ Furthermore, NHW adults with CRS were more likely to receive care from an additional specialist (ie, an allergist) and were more likely to undergo surgical treatment at a private hospital than other racial and ethnic groups.^5^, ^23^ In contrast, one study at a single academic center reported that patients with CRS who presented for tertiary rhinology services were the same race/ethnicity as the general hospital population and that there were no racial or ethnic differences in presenting SNOT‐22 scores for patients.^28^

NHB and Hispanic patients with CRS were more likely to delay medical care due to financial insecurity, and Hispanic patients were more likely to be uninsured compared to NHB, NHW, and Asian patients.^5^ NHB patients had fewer follow‐up appointments and reportedly received less antibiotic or surgical treatment despite having higher average SNOT‐22 scores.^30^ Hispanic ethnicity was associated with delayed time to sinus surgery, independent of disease severity.^31^ One study reported that Asian patients with CRS were significantly less likely to elect for ESS compared to non‐Asian patients.^32^ Anatomical differences in maxillary sinus (MS) size and shape were also associated with disparities in CRS susceptibility, with Asian patients having taller MS and greater ostium distance, potentially impairing mucociliary clearance.^33^

Racial disparities were independently associated with the severity of CRS symptoms and radiographic/endoscopic findings.^15^ Non‐white patients had higher average SNOT‐22 scores, more nasal polyps, and required more extensive ESS compared to NHW patients.^26^, ^34^ Specifically, NHB patients had worse SNOT‐22 scores compared to NHW and Hispanic patients following medical and surgical treatment during long‐term follow‐up.^15^, ^16^, ^30^ NHB and Hispanic patients reported more severe baseline symptoms but showed greater improvement at follow‐up visits compared to NHW patients.^15^, ^18^ In patients with cystic fibrosis and CRS, Hispanic ethnicity correlated with greater sinonasal symptom burden.^27^

### Gender

Nine studies (34.6%) discussed how patient gender contributed to health disparities in CRS specifically regarding patient presentation, symptom burden, and treatment outcome. Disease presentation differed between males and females, including how male patients were more likely to present with CRS with nasal polyps (CRSwNP) compared to females.^35^, ^36^, ^37^ Female patients were reported to have a higher incidence of comorbid conditions including asthma and depression compared to males.^35^, ^37^

Symptom burden, characterized by SNOT‐22, QoL, and Rhinosinusitis Disability Index scores, was found to be significantly higher in female patients compared to male patients in both the preoperative and postoperative period.^6^, ^35^, ^36^, ^37^, ^38^ Overall QoL was self‐reported to be worse in females than males, despite male patients exhibiting worse objective preoperative scores.^6^, ^27^, ^35^, ^36^, ^37^, ^38^ One prospective study similarly reported that females experienced decreased QoL subjectively, while males showed more severe objective clinical measures such as sinus computed tomography (CT; Lund‐Mackay scores) and nasal endoscopy (Lund‐Kennedy scores).^35^ In contrast, another study suggested that CRS was not more prevalent amongst females based on demographic data collected in a tertiary rhinology clinic compared to the hospital‐wide populations.^21^

Reports of gender‐related treatment outcomes differed among studies. Outcomes such as olfactory improvement and reduction in inflammation after medical or surgical treatment were similar between genders.^6^, ^27^, ^29^, ^38^ Male patients with CRSwNP had more frequent recurrences postoperatively.^35^, ^36^ One study described changes in monetized productivity loss after ESS between genders and highlighted greater productivity loss amongst females than males.^22^

### Age

Four studies (15.4%) discussed age‐related differences in CRS patients that influenced symptom severity, treatment outcomes, and adherence to follow‐up care. Symptom severity at initial presentation, quantified by SNOT‐22 scores, was described in three of these studies.^6^, ^23^, ^27^ One study reported no significant association between age and SNOT‐22 scores^27^; however, another found that increasing age was associated with lower preoperative SNOT‐22 scores.^6^

One study assessed differences in patient presentation at a private and public hospital and reported that the mean age was similar between hospitals, but the distribution of ages differed, with more middle‐aged CRS patients (40‐59 years) presenting at the public hospital and younger patients (<39 years) at the private hospital.^23^ Lastly, one study described that patients with CRS were, on average, 7 years older than the general hospital population.^21^

Treatment outcomes were also found to be associated with age. Increased age was associated with lower Lund‐Mackay scores at presentation.^27^ Younger age was associated with a greater likelihood of achieving a clinically significant improvement in postoperative SNOT‐22 scores.^6^ Lastly, younger patients were found to be more likely lost to postoperative follow‐up.^6^

### Geographic Region

Four studies (15.4%) discussed geographic region as a source of disparity in CRS. Exposure to air pollution varied by geographic location and correlated with CRS disease severity and prevalence, disproportionately affecting lower SES populations.^17^ Individuals who lived in regions with higher levels of air pollution experienced increased rates of CRSwNP and required greater healthcare utilization for CRS management.^17^ One study demonstrated that patients from regions of geographic socioeconomic deprivation experienced more severe disease and inferior outcomes following ESS.^4^ Patients in rural or underserved areas were also less likely to undergo ESS at high‐volume centers. If they did undergo ESS at lower‐volume centers, this population was associated with inferior postoperative outcomes and higher rates of revision surgery compared to patients who had surgery at high‐volume centers.^4^

Similarly, another study showed how geographic regions intersected with SES to impact CRS treatment modalities and severity.^24^ It was noted that individuals residing in regions with limited specialist access often relied on primary care management for sinonasal symptoms, resulting in delayed treatment escalation and increased disease burden.^24^

Additionally, in the pediatric population, another study demonstrated that children in rural areas had less access to advanced rhinologic care and were more likely to experience delays in initial diagnosis compared to their urban counterparts.^29^

### Suggested Solutions

Of the 26 studies analyzed in this review, only three proposed actionable steps to reduce disparities. Smith et al proposed the creation of a nationally representative pediatric CRS database for the purpose of evaluating the social and environmental influences on pediatric sinus health in order to mitigate disparities.^29^ Pandrangi et al recommended that national databases that collect data on adult sinusitis patients should more objectively define chronic versus acute rhinosinusitis as well as treatments for sinusitis in order to better characterize disease.^25^ Lastly, Behnke et al suggested that higher‐level studies are necessary to control for gender‐related factors to better understand the QoL differences between female and male patients with CRS.^36^

## Discussion

Health disparities in CRS are influenced by a range of social determinants including SES, race/ethnicity, gender, age, and geographic location. The majority of studies included in this review reported that SES was a key determinant for CRS presentation and outcomes, with lower‐income patients and those covered by public insurance experiencing more severe symptoms, reduced follow‐up care, and increased financial barriers to treatment. Conversely, patients with higher incomes or private insurance demonstrated better posttreatment outcomes following ESS. Disparities in care were also observed in pediatric populations, where children from lower SES backgrounds had poorer QoL; however, SES did not predict the type of treatment received. Together, these findings suggest that CRS patients with a lower SES or who have public health insurance may tend to delay their care secondary to financial barriers, contributing to the higher degree of disease severity on presentation noted within this patient population.^39^ Furthermore, once these patients do seek care, studies clearly demonstrate they are more prone to irregular follow‐up, worsening their long‐term outcomes. These findings highlight the critical role of financial stability and access to resources in determining CRS severity and long‐term outcomes. Interventions that reduce financial obstacles to care, such as improving access to affordable medications and follow‐up care via assistance programs, may be essential for mitigating the impact of SES‐related disparities.

Racial and ethnic disparities in CRS care were also evident. It was reported that NHB and Hispanic patients experienced greater disease severity at baseline, but presented for fewer follow‐ups, fewer surgical interventions, and experienced poorer long‐term outcomes compared to NHW patients. Anatomical differences, such as variations in sinus structure in Asian patients, were also associated with CRS susceptibility and symptom clearance, suggesting that biological factors may intersect with social determinants to influence disease outcomes. Gender differences were observed in symptom burden and QoL, with women reporting worse subjective outcomes on SNOT‐22 questionnaires despite men exhibiting more severe objective clinical measures. Interestingly, it has been generally accepted in the literature that men are more likely to have CRSwNP and women are more likely to have chronic rhinosinusitis without nasal polyps, which adds more complexity to these gender‐based discrepancies in subjective symptoms.^36^, ^40^, ^41^ Additionally, the SNOT‐22 survey evaluates for nonspecific symptoms including headache, fatigue, and feeling sad/embarrassed about symptoms, which may be a confounder in high overall scores even in the absence of CRS.^42^, ^43^ It is also hypothesized that variations in the sinus microbiome by gender may exist, which could drive the differential disease burden and symptomatology in men versus women, but additional research is required in this field.^44^

Age and geographic location were reported as contributors to disparities. It was described that younger patients experienced greater symptomatic improvement in the postoperative period but had lower adherence to long‐term follow‐up. Older patients (>55 years) did not experience the same level of improvement in olfaction scores in the postoperative period compared to their younger counterparts. One potential explanation for why this population had a lesser degree of improvement in olfaction is that older patients more commonly reported poorer baseline olfaction before treatment.^45^ Further, older patients may have less olfactory reserve at baseline or loss of olfactory neuroepithelium with age or disease‐related changes. Regarding geographic location, patients from rural or underserved areas had more severe disease, delayed diagnosis, and suboptimal treatment outcomes compared to patients from urban locations. Distance to pharmacy and treating hospital is known to play a large role in disease progression and is often inversely related to severity.^4^, ^24^ Another large contributor to disease severity was air pollution, which was shown to be geography dependent. Exposure to air pollution reportedly had a disproportionate effect on lower SES patients and led to more severe CRS symptoms and inferior treatment outcomes. These important findings underscore the multifaceted nature of health disparities in CRS and suggest that tailored interventions addressing both social and biological factors are necessary to improve equity in CRS care, including policies to reduce emissions and enhance green spaces.

Previous work has demonstrated that the demographics of participants in prospective clinical trials for CRS contained significant disparities compared to US census data including the underrepresentation of NHB, Asian, Hispanic, and Native American patients, as well as females.^14^, ^46^ These findings suggest the need for greater inclusion of diverse populations in rhinology clinical trials, which are often industry‐sponsored and inform FDA approvals. Addressing these gaps is critical to ensuring therapeutic efficacy and improving understanding of CRSwNP endotypes, given the reliance on patient‐reported outcomes in treatment evaluation.

This review represents, to the best of our knowledge, the most comprehensive review of health disparities in CRS to date. No previous study has involved experienced medical librarians who searched multiple reputable databases with controlled vocabularies, keyword searches, and no limitations on year published. Moreover, the inclusion of a dual‐author independent study screening process adds reliability to the selection of relevant studies. The detailed extraction of diverse health disparity outcomes and population characteristics, organized into a structured table, further strengthens our study's utility in comprehensively analyzing health disparities in CRS. This robust approach highlights our commitment to capturing a nuanced understanding of health inequities within the United States.

However, this review is subject to several limitations that may influence its findings and interpretations. One limitation is the subjective nature of categorizing studies, as many examined overlapping themes related to disparities in CRS, which may have introduced bias in the thematic organization of the review. Another significant limitation is the use of self‐reported race as a variable. While commonly utilized, self‐reported race is an inherently imperfect metric with loose associations to genetic ancestry and should not be considered definitive of biological composition. Furthermore, this review focused primarily on NHW, NHB, and Hispanic patients due to the larger volume of literature addressing these populations. Less literature focused on Asian patients, and no literature included Native American patients. This imbalance highlights the need for more inclusive research to better understand the experiences of underrepresented populations. Finally, the scope and scale of the cited studies varied. Some focused on disparity trends within a single institution, whereas others analyzed broader patient populations, affecting the generalizability of findings to the general population. Despite these limitations, this review offers insight into the multifactorial contributors to disparities in CRS. By emphasizing key areas of disparity and highlighting gaps in current research, this study may act as a foundation for future research aimed at addressing inequities in CRS outcomes.

The goal of this review was to provide transparency to the current state of literature in health disparities in CRS and elucidate their findings and implications. Addressing each health disparity population discussed in this review with an emphasis on the SDOH has the potential to significantly enhance outcomes for all patients with CRS. Only 3 out of the 26 studies analyzed in this review proposed actionable steps to reduce disparities. Additional sociodemographic factors that may be relevant to consider for future research include the presence of community/social support, literacy level, availability of transportation, and access to safe drinking water. Future work should also focus on creating targeted interventions to address disparities in access to care, treatment compliance, and health outcomes based on SES, race/ethnicity, gender, age, and geographic region.

## Conclusion

Existing literature on disparities in CRS primarily highlights the significant impact of SES and race on disease presentation, management, and progression. These studies reveal that individuals from lower SES backgrounds or racially marginalized groups often face delayed diagnoses, limited access to specialty care, and suboptimal outcomes. However, disparities extend beyond SES and race, encompassing factors such as gender, age, and geographic region. These include variations in CRS prevalence and treatment patterns based on gender and age‐specific factors and geographic barriers in rural versus urban settings. Despite these findings, there remains a substantial gap in understanding the root causes of these disparities. Further research should aim to investigate these underlying etiologies of disparities, such as systemic healthcare inequities, implicit bias, and environmental exposures, while prioritizing the development of actionable solutions. By addressing disparities, the goal of achieving equitable care for all CRS patients can be advanced.

## Author Contributions

**Russell A. Whitehead**, Participated in study conceptualization, data collection, drafting and editing the manuscript, approved the final manuscript, and takes full responsibility for its content. **Abdel R. Metwally**, Participated in study conceptualization, data collection, drafting and editing the manuscript, approved the final manuscript, and takes full responsibility for its content. **Evan A. Patel**, Participated in study conceptualization, data collection, drafting and editing the manuscript, approved the final manuscript, and takes full responsibility for its content. **Thomas Cyberski**, Participated in study conceptualization, drafting and editing the manuscript, approved the final manuscript, and takes full responsibility for its content. **Robin Powszok**, Participated in study conceptualization, drafting and editing the manuscript, approved the final manuscript, and takes full responsibility for its content. **Peter Filip**, Participated in drafting and editing the manuscript, approved the final manuscript, and takes full responsibility for its content. **Peter Papagiannopoulos**, Participated in drafting and editing the manuscript, approved the final manuscript, and takes full responsibility for its content. **Bobby A. Tajudeen**, Participated in drafting and editing the manuscript, approved the final manuscript, and takes full responsibility for its content. **Pete S. Batra**, Participated in study conceptualization, drafting and editing the manuscript, approved the final manuscript, and takes full responsibility for its content.

## Disclosures

### Competing interests

The authors declare no conflicts of interest.

### Funding source

None.

## Supporting information


**Supplemental File S1.** Full search strategy crafted by librarians.
