# Supplementary material for: A Systematic Review of Health Disparities in Chronic Rhinosinusitis in the United States
Source: OTO Open. 2025 Sep 12;9(3):e70163. doi: 10.1002/oto2.70163 (PMC12426902; doi:10.1002/oto2.70163)
Supplement: Supplementary file 1 — Supplemental File S1. Full search strategy crafted by librarians. [file OTO2-9-e70163-s001.docx]

Search Strategy

By Jennifer C Westrick, MSLIS, AHIP  and Lorena Juarez, MLIS

Run 8-30; re-run 9-3-2024

A comprehensive literature search was developed by the authors and was run by experienced medical librarian (JW) on Sept 3, 2024 in the following databases:  PubMed/MEDLINE, Embase, CINAHL, Scopus, and the Cochrane CENTRAL Register of Controlled Trials.  Both controlled vocabularies (e.g., MeSH terms) and keywords in the title or abstract fields were searched.  There were no restrictions on the age of participants or language of publication. Additionally, a hand search was conducted of the reference lists of selected articles. Reproducible search strategies are attached – see Appendix ()

**PubMed**

((("Healthcare Disparities"[Mesh] OR "Health Status Disparities"[Mesh] OR "Health Services Accessibility"[Mesh] OR "Health Inequities"[Mesh] OR "Social determinants of health"[Mesh])

OR ((Disparit*[Title/Abstract] OR determinant*[Title/Abstract] OR discriminat*[Title/Abstract] OR equit*[Title/Abstract] OR inequalit*[Title/Abstract] OR unequal[Title/Abstract])

AND (age[Title/Abstract] OR African*[Title/Abstract] OR alcohol[Title/Abstract] OR Appalachian[Title/Abstract] OR age[Title/Abstract] OR Asian[Title/Abstract] OR barrier*[Title/Abstract] OR black*[Title/Abstract] OR Caucasian*[Title/Abstract] OR demographic*[Title/Abstract] OR disabilit*[Title/Abstract] OR education*[Title/Abstract] OR employ*[Title/Abstract] OR ethnic*[Title/Abstract] OR expos*[Title/Abstract] OR gender[Title/Abstract] OR geograph*[Title/Abstract] OR health[Title/Abstract] OR Hispanic*[Title/Abstract] OR hous*[Title/Abstract] OR "health literacy"[Title/Abstract] OR income[Title/Abstract] OR indigenous[Title/Abstract] OR insurance[Title/Abstract] OR insured[Title/Abstract] OR "low income"[Title/Abstract] OR Latin*[Title/Abstract] OR Medicaid[Title/Abstract] OR Medicare[Title/Abstract] OR "mental health"[Title/Abstract] OR "mental illness"[Title/Abstract] OR "mental well-being"[Title/Abstract] OR “Native American”[Title/Abstract] OR “Pacific Islander”[Title/Abstract] OR "patient access"[Title/Abstract] OR poverty[Title/Abstract] OR "payer type"[Title/Abstract] OR race[Title/Abstract] OR racial[Title/Abstract] OR rural*[Title/Abstract] OR "travel time"[Title/Abstract] OR sex[Title/Abstract] OR sociodemographic[Title/Abstract] OR socio-demographic[Title/Abstract] OR socioeconomic[Title/Abstract] OR socio-economic[Title/Abstract] OR substance abuse[Title/Abstract] OR tobacco[Title/Abstract] OR underinsured[Title/Abstract] OR underserved[Title/Abstract] OR unemployed[Title/Abstract] OR uninsured[Title/Abstract] OR urban[Title/Abstract] OR white[Title/Abstract] OR young*[Title/Abstract])))

AND ((("Chronic rhinosinusitis"[Title/Abstract] OR "Chronic sinusitis"[Title/Abstract] OR "Chronic rhinitis"[Title/Abstract] OR Rhinitis[Title/Abstract] OR Sinusitis[Title/Abstract] OR Rhinosinusitis[Title/Abstract] OR "Sinonasal disease"[Title/Abstract] OR "Non-allergic rhinitis"[Title/Abstract] OR "Nasal polyp*"[Title/Abstract] OR "Sinusitis"[Mesh])))

NOT (("allergic rhinitis" NOT ("chronic rhinosinusitis" OR rhinosinusitis OR nonallergic OR non-allergic))))

AND ("United States"[MeSH Terms] OR "United States" OR "USA" OR America* OR "AL" OR "AK" OR "AZ" OR "AR" OR "CA" OR "CO" OR "CT" OR "DE" OR "FL" OR "GA" OR "HI" OR "ID" OR "IL" OR "IA" OR "KS" OR "KY" OR "LA" OR "ME" OR "MA" OR "MI" OR "MN" OR "MS" OR "MO" OR "MT" OR "NE" OR "NV" OR "NH" OR "NJ" OR "NM" OR "NY" OR "NC" OR "ND" OR "OH" OR "OK" OR "PA" OR "RI" OR "TN" OR "TX" OR "UT" OR "VT" OR "VA" OR "WA" OR "WV" OR "WI" OR "WY" OR Alabama OR Alaska OR Arizona OR Arkansas OR California OR Colorado OR Connecticut OR Delaware OR Florida OR Georgia OR Hawaii OR Idaho OR Illinois OR Indiana OR Iowa OR Kansas OR Kentucky OR Louisiana OR Maine OR Maryland OR Massachusetts OR Michigan OR Minnesota OR Mississippi OR Missouri OR Montana OR Nebraska OR Nevada OR "New Hampshire" OR "New Jersey" OR "New Mexico" OR "New York" OR "North Carolina" OR "North Dakota" OR Ohio OR Oklahoma OR Oregon OR Pennsylvania OR "Rhode Island" OR "South Carolina" OR "South Dakota" OR Tennessee OR Texas OR Utah OR Vermont OR Virginia OR Washington OR "West Virginia" OR Wisconsin OR Wyoming OR Appalachia* OR "Great Lakes" OR Medicare OR Medicaid OR "Mid-Atlantic" OR Midwest* or "New England" or Northwest* OR Southeast* OR Southwest*)

**Embase**

('health care disparity'/exp OR 'health disparity'/exp OR 'health care access'/exp

OR ((disparit*:ab,ti OR determinant*:ab,ti OR discriminat*:ab,ti OR equit*:ab,ti OR inequalit*:ab,ti OR unequal:ab,ti)

AND (african*:ab,ti OR alcohol:ab,ti OR appalachian:ab,ti OR age:ab,ti OR asian:ab,ti OR barrier*:ab,ti OR black*:ab,ti OR caucasian*:ab,ti OR demographic*:ab,ti OR disabilit*:ab,ti OR education*:ab,ti OR employ*:ab,ti OR ethnic*:ab,ti OR expos*:ab,ti OR gender:ab,ti OR geograph*:ab,ti OR health:ab,ti OR hispanic*:ab,ti OR hous*:ab,ti OR 'health literacy':ab,ti OR income:ab,ti OR indigenous:ab,ti OR insurance:ab,ti OR insured:ab,ti OR 'low income':ab,ti OR latin*:ab,ti OR medicaid:ab,ti OR medicare:ab,ti OR 'mental health':ab,ti OR 'mental illness':ab,ti OR 'mental well-being':ab,ti OR 'native american':ab,ti OR 'pacific islander':ab,ti OR 'patient access':ab,ti OR poverty:ab,ti OR 'payer type':ab,ti OR race:ab,ti OR racial:ab,ti OR rural*:ab,ti OR 'travel time':ab,ti OR sex:ab,ti OR sociodemographic:ab,ti OR 'socio demographic':ab,ti OR socioeconomic:ab,ti OR 'socio economic':ab,ti OR 'substance abuse':ab,ti OR tobacco:ab,ti OR underinsured:ab,ti OR underserved:ab,ti OR unemployed:ab,ti OR uninsured:ab,ti OR urban:ab,ti OR white:ab,ti OR young*:ab,ti)))

AND ('chronic rhinosinusitis':ab,ti OR 'chronic sinusitis':ab,ti OR 'chronic rhinitis':ab,ti OR rhinitis:ab,ti OR sinusitis:ab,ti OR rhinosinusitis:ab,ti OR 'sinonasal disease':ab,ti OR 'non-allergic rhinitis':ab,ti OR 'nasal polyp':ab,ti)

NOT (('allergic rhinitis'/exp OR 'allergic rhinitis') NOT ('chronic rhinosinusitis'/exp OR 'chronic rhinosinusitis' OR 'rhinosinusitis'/exp OR rhinosinusitis OR nonallergic OR 'non allergic'))

AND ('united states' OR 'usa' OR america* OR 'al' OR 'ak' OR 'az' OR 'ar' OR 'ca' OR 'co' OR 'ct' OR 'fl' OR 'ga' OR 'hi' OR 'id' OR 'il' OR 'ia' OR 'ks' OR 'ky' OR 'la' OR 'me' OR 'ma' OR 'mi' OR 'mn' OR 'ms' OR 'mo' OR 'mt' OR 'ne' OR 'nv' OR 'nh' OR 'nj' OR 'nm' OR 'ny' OR 'nc' OR 'nd' OR 'oh' OR 'ok' OR 'pa' OR 'ri' OR 'tn' OR 'tx' OR 'ut' OR 'vt' OR 'va' OR 'wa' OR 'wv' OR 'wi' OR 'wy' OR alabama OR alaska OR arizona OR arkansas OR california OR colorado OR connecticut OR delaware OR florida OR georgia OR hawaii OR idaho OR illinois OR indiana OR iowa OR kansas OR kentucky OR louisiana OR maine OR maryland OR massachusetts OR michigan OR minnesota OR mississippi OR missouri OR montana OR nebraska OR nevada OR 'new hampshire' OR 'new jersey' OR 'new mexico' OR 'new york' OR 'north carolina' OR 'north dakota' OR ohio OR oklahoma OR oregon OR pennsylvania OR 'rhode island' OR 'south carolina' OR 'south dakota' OR tennessee OR texas OR utah OR vermont OR virginia OR washington OR 'west virginia' OR wisconsin OR wyoming OR appalachia* OR 'great lakes' OR medicare OR medicaid OR 'mid-atlantic' OR midwest* OR 'new england' OR northwest* OR southeast* OR southwest*)

**CINAHL**

( (MH "Health Status")

OR ( ( TI ( Disparit* OR determinant* OR discriminat* OR equit* OR inequalit* OR unequal )

OR AB ( Disparit* OR determinant* OR discriminat* OR equit* OR inequalit* OR unequal ) )

AND ( TI ( age OR African* OR alcohol OR Appalachian OR age OR Asian OR barrier* OR black* OR Caucasian* OR demographic* OR disabilit* OR education* OR employ* OR ethnic* OR expos* OR gender OR geograph* OR health OR Hispanic* OR hous* OR "health literacy" OR income OR indigenous OR insurance OR insured OR "low income" OR Latin* OR Medicaid OR Medicare OR "mental health" OR "mental illness" OR "mental well-being" OR “Native American” OR “Pacific Islander” OR "patient access" OR poverty OR "payer type" OR race OR racial OR rural* OR "travel time" OR sex OR sociodemographic OR socio-demographic OR socioeconomic OR socio-economic OR substance abuse OR tobacco OR underinsured OR underserved OR unemployed OR uninsured OR urban OR white OR young* )

OR AB ( age OR African* OR alcohol OR Appalachian OR age OR Asian OR barrier* OR black* OR Caucasian* OR demographic* OR disabilit* OR education* OR employ* OR ethnic* OR expos* OR gender OR geograph* OR health OR Hispanic* OR hous* OR "health literacy" OR income OR indigenous OR insurance OR insured OR "low income" OR Latin* OR Medicaid OR Medicare OR "mental health" OR "mental illness" OR "mental well-being" OR “Native American” OR “Pacific Islander” OR "patient access" OR poverty OR "payer type" OR race OR racial OR rural* OR "travel time" OR sex OR sociodemographic OR socio-demographic OR socioeconomic OR socio-economic OR substance abuse OR tobacco OR underinsured OR underserved OR unemployed OR uninsured OR urban OR white OR young* ) ) ) )

AND ( TI ( "Chronic rhinosinusitis" OR "Chronic sinusitis" OR "Chronic rhinitis" OR Rhinitis OR Sinusitis OR Rhinosinusitis OR "Sinonasal disease" OR "Non-allergic rhinitis" OR "Nasal polyp" ) OR AB ( "Chronic rhinosinusitis" OR "Chronic sinusitis" OR "Chronic rhinitis" OR Rhinitis OR Sinusitis OR Rhinosinusitis OR "Sinonasal disease" OR "Non-allergic rhinitis" OR "Nasal polyp" ) )

NOT ( "allergic rhinitis" NOT ("chronic rhinosinusitis" OR rhinosinusitis OR nonallergic OR non-allergic) )

**Scopus**

TITLE-ABS ( ( ( disparit* OR determinant* OR discriminat* OR equit* OR inequalit* OR unequal ) AND ( age OR african* OR alcohol OR appalachian OR age OR asian OR barrier* OR black* OR caucasian* OR demographic* OR disabilit* OR education* OR employ* OR ethnic* OR expos* OR gender OR geograph* OR health OR hispanic* OR hous* OR "health literacy" OR income OR indigenous OR insurance OR insured OR "low income" OR latin* OR medicaid OR medicare OR "mental health" OR "mental illness" OR "mental well-being" OR "Native American" OR "Pacific Islander" OR "patient access" OR poverty OR "payer type" OR race OR racial OR rural* OR "travel time" OR sex OR sociodemographic OR socio-demographic OR socioeconomic OR socio-economic OR "substance abuse" OR tobacco OR underinsured OR underserved OR unemployed OR uninsured OR urban OR white OR young* ) ) ) )

AND ( TITLE-ABS ( "Chronic rhinosinusitis" OR "Chronic sinusitis" OR "Chronic rhinitis" OR rhinitis OR sinusitis OR rhinosinusitis OR "Sinonasal disease" OR "Non-allergic rhinitis" OR "Nasal polyp" ) )

AND NOT ( ( "allergic rhinitis" AND NOT ( "chronic rhinosinusitis" OR rhinosinusitis OR nonallergic OR non-allergic ) ) )

AND ( "United States" OR "USA" OR america* OR "AL" OR "AK" OR "AZ" OR "AR" OR "CA" OR "CO" OR "CT" OR "DE" OR "FL" OR "GA" OR "HI" OR "ID" OR "IL" OR "IA" OR "KS" OR "KY" OR "LA" OR "ME" OR "MA" OR "MI" OR "MN" OR "MS" OR "MO" OR "MT" OR "NE" OR "NV" OR "NH" OR "NJ" OR "NM" OR "NY" OR "NC" OR "ND" OR "OH" OR "OK" OR "PA" OR "RI" OR "TN" OR "TX" OR "UT" OR "VT" OR "VA" OR "WA" OR "WV" OR "WI" OR "WY" OR alabama OR alaska OR arizona OR arkansas OR california OR colorado OR connecticut OR delaware OR florida OR georgia OR hawaii OR idaho OR illinois OR indiana OR iowa OR kansas OR kentucky OR louisiana OR maine OR maryland OR massachusetts OR michigan OR minnesota OR mississippi OR missouri OR montana OR nebraska OR nevada OR "New Hampshire" OR "New Jersey" OR "New Mexico" OR "New York" OR "North Carolina" OR "North Dakota" OR ohio OR oklahoma OR oregon OR pennsylvania OR "Rhode Island" OR "South Carolina" OR "South Dakota" OR tennessee OR texas OR utah OR vermont OR virginia OR washington OR "West Virginia" OR wisconsin OR wyoming OR appalachia* OR "Great Lakes" OR medicare OR medicaid OR "Mid-Atlantic" OR midwest* OR "New England" OR northwest* OR southeast* OR southwest* )

**Cochrane CENTRAL Register of Controlled Trials**

((Disparit* OR determinant* OR discriminat* OR equit* OR inequalit* OR unequal)

AND (age OR African* OR alcohol OR Appalachian OR age OR Asian OR barrier* OR black* OR Caucasian* OR demographic* OR disabilit* OR education* OR employ* OR ethnic* OR expos* OR gender OR geograph* OR health OR Hispanic* OR hous* OR "health literacy" OR income OR indigenous OR insurance OR insured OR "low income" OR Latin* OR Medicaid OR Medicare OR "mental health" OR "mental illness" OR "mental well-being" OR “Native American” OR “Pacific Islander” OR "patient access" OR poverty OR "payer type" OR race OR racial OR rural* OR "travel time" OR sex OR sociodemographic OR socio-demographic OR socioeconomic OR socio-economic OR “substance abuse” OR tobacco OR underinsured OR underserved OR unemployed OR uninsured OR urban OR white OR young*))

AND ((("Chronic rhinosinusitis" OR "Chronic sinusitis" OR "Chronic rhinitis" OR Rhinitis OR Sinusitis OR Rhinosinusitis OR "Sinonasal disease" OR "Non-allergic rhinitis" OR "Nasal polyp”))))

NOT (("allergic rhinitis"

NOT ("chronic rhinosinusitis" OR rhinosinusitis OR nonallergic OR non-allergic))))

AND ("United States" OR "USA" OR America* OR "AL" OR "AK" OR "AZ" OR "AR" OR "CA" OR "CO" OR "CT" OR "DE" OR "FL" OR "GA" OR "HI" OR "ID" OR "IL" OR "IA" OR "KS" OR "KY" OR "LA" OR "ME" OR "MA" OR "MI" OR "MN" OR "MS" OR "MO" OR "MT" OR "NE" OR "NV" OR "NH" OR "NJ" OR "NM" OR "NY" OR "NC" OR "ND" OR "OH" OR "OK" OR "PA" OR "RI" OR "TN" OR "TX" OR "UT" OR "VT" OR "VA" OR "WA" OR "WV" OR "WI" OR "WY" OR Alabama OR Alaska OR Arizona OR Arkansas OR California OR Colorado OR Connecticut OR Delaware OR Florida OR Georgia OR Hawaii OR Idaho OR Illinois OR Indiana OR Iowa OR Kansas OR Kentucky OR Louisiana OR Maine OR Maryland OR Massachusetts OR Michigan OR Minnesota OR Mississippi OR Missouri OR Montana OR Nebraska OR Nevada OR "New Hampshire" OR "New Jersey" OR "New Mexico" OR "New York" OR "North Carolina" OR "North Dakota" OR Ohio OR Oklahoma OR Oregon OR Pennsylvania OR "Rhode Island" OR "South Carolina" OR "South Dakota" OR Tennessee OR Texas OR Utah OR Vermont OR Virginia OR Washington OR "West Virginia" OR Wisconsin OR Wyoming OR Appalachia* OR "Great Lakes" OR Medicare OR Medicaid OR "Mid-Atlantic" OR Midwest* or "New England" or Northwest* OR Southeast* OR Southwest*)
